# Assessment of cerebral drug occupancy in humans using a single PET-scan: A [^11^C]UCB-J PET study

**DOI:** 10.1007/s00259-024-06759-x

**Published:** 2024-05-17

**Authors:** Maja R. Marstrand-Joergensen, Gjertrud L. Laurell, Susan Herrmann, Arafat Nasser, Annette Johansen, Anton Lund, Thomas L. Andersen, Gitte M. Knudsen, Lars H. Pinborg

**Affiliations:** 1 Epilepsy Clinic, Copenhagen University Hospital Rigshospitalet, Blegdamsvej 9, Copenhagen O, 2100 Denmark; 2 Neurobiology Research Unit, Copenhagen University Hospital Rigshospitalet, Rigshospitalet, Building 8057, Blegdamsvej 9, Copenhagen, 8057, DK-2100 Denmark; 3 Department of Neurology, Copenhagen University Hospital Rigshospitalet, Copenhagen, 2100 Denmark; 4 Faculty of Health and Medical Sciences, University of Copenhagen, Copenhagen, Denmark; 5 Molecular Imaging and Neuropathology Area, New York State Psychiatric Institute, New York, NY USA; 6 Department of Psychiatry, Columbia University, New York, NY USA; 7 Department of Clinical Biochemistry, Copenhagen University Hospital Rigshospitalet, Glostrup, Denmark; 8 Department of Neuroanaesthesiology, Copenhagen University Hospital Rigshospitalet, Copenhagen, 2100 Denmark; 9 Department of Clinical Physiology & Nuclear Medicine, Copenhagen University Hospital Rigshospitalet, Copenhagen, 2100 Denmark; 10 Department of Clinical Medicine, Faculty of Health and Medicine, University of Copenhagen, Copenhagen, Denmark

**Keywords:** PET kinetic modeling, Displacement, Levetiracetam, Occupancy, Half maximal inhibitory concentration, Healthy volunteers

## Abstract

**Purpose:**

Here, we evaluate a PET displacement model with a Single-step and Numerical solution in healthy individuals using the synaptic vesicle glycoprotein (SV2A) PET-tracer [^11^C]UCB-J and the anti-seizure medication levetiracetam (LEV). We aimed to (1) validate the displacement model by comparing the brain LEV-SV2A occupancy from a single PET scan with the occupancy derived from two PET scans and the Lassen plot and (2) determine the plasma LEV concentration-SV2A occupancy curve in healthy individuals.

**Methods:**

Eleven healthy individuals (five females, mean age 35.5 [range: 25–47] years) underwent two 120-min [^11^C]UCB-J PET scans where an LEV dose (5–30 mg/kg) was administered intravenously halfway through the first PET scan to partially displace radioligand binding to SV2A. Five individuals were scanned twice on the same day; the remaining six were scanned once on two separate days, receiving two identical LEV doses. Arterial blood samples were acquired to determine the arterial input function and plasma LEV concentrations. Using the displacement model, the SV2A-LEV target engagement was calculated and compared with the Lassen plot method. The resulting data were fitted with a single-site binding model.

**Results:**

SV2A occupancies and V_ND_ estimates derived from the displacement model were not significantly different from the Lassen plot (*p* = 0.55 and 0.13, respectively). The coefficient of variation was 14.6% vs. 17.3% for the Numerical and the Single-step solution in Bland-Altman comparisons with the Lassen plot. The average half maximal inhibitory concentration (IC_50_), as estimated from the area under the curve of the plasma LEV concentration, was 12.5 µg/mL (95% CI: 5–25) for the Single-Step solution, 11.8 µg/mL (95% CI: 4–25) for the Numerical solution, and 6.3 µg/mL (95% CI: 0.08-21) for the Lassen plot. Constraining Emax to 100% did not significantly improve model fits.

**Conclusion:**

Plasma LEV concentration vs. SV2A occupancy can be determined in humans using a single PET scan displacement model. The average concentration of the three computed IC_50_ values ranges between 6.3 and 12.5 µg/mL. The next step is to use the displacement model to evaluate LEV occupancy and corresponding plasma concentrations in relation to treatment efficacy.

**Clinical trial registration:**

NCT05450822. Retrospectively registered 5 July 2022 https://clinicaltrials.gov/ct2/results? term=NCT05450822&Search=Search.

**Supplementary Information:**

The online version contains supplementary material available at 10.1007/s00259-024-06759-x.

## Introduction

About 1% of the global population suffers from epilepsy [1], and for approximately 30% of the patients, anti-seizure medication (ASM) does not lead to seizure freedom [2]. Levetiracetam (LEV) is a very commonly used ASM approved for the treatment of various types of epilepsy [3]. Both [^11^C]UCB-J and LEV bind to the synaptic vesicle glycoprotein 2 A (SV2A) [4, 5] and prior studies have demonstrated displacement of [^11^C]UCB-J with LEV [6–9]. SV2A is located in vesicles of the presynaptic nerve terminal [10] and is recognized as a synaptic marker [11]. Levetiracetam binds to several targets: α-amino-3-hydroxy-5-methyl-4-isoxazolepropionic acid receptors (AMPA-receptors), high-voltage-activated calcium channels, and the GABAergic system [12, 13]. In contrast, the ASM brivaracetam (BRIVA) is a selective SV2A ligand [14, 15]. Interestingly, a reduction in synaptic density has been demonstrated in patients with mesial temporal lobe epilepsy [11], and in the seizure onset zone, [^11^C]UCB-J binding is more reduced than [^18^F]FDG PET is [16]. Examining the impact of LEV on cerebral SV2A binding presents an opportunity to deepen our insights into the relationship between synaptic density, epilepsy, and the response to LEV treatment in individuals with epilepsy.

A previous study has investigated the plasma LEV-brain SV2A occupancy in healthy individuals employing a two-parameter Emax model and demonstrated an Emax of 88.4% by displacing [^11^C]UCB-J with a small dose range (250, 600, and 1500 mg) of LEV [6]. This LEV Emax was significantly different from 100% [6]. In comparison, BRIVA (dose range 50, 100, 200 mg) was able to block 100% of the tracer binding [6].

We have recently presented a PET kinetic model that estimates drug occupancy from a single PET displacement scan after a pharmacological intervention with BRIVA [17]. The model builds on a modified one-tissue compartment model (1TCM) with a time-dependent occupancy term and two distinct solutions: an approximate analytical Single-step solution characterized by a step function leading to maximal drug occupancy and a Numerical solution employing Euler Forward propagation and a continuous occupancy function [17]. The Numerical solution was initially developed for more slow-acting drugs, where maximal occupancy was not anticipated to occur instantly [17]. The performance of this newly developed displacement model was assessed in simulated data and pig PET scans where an arterial input function is available. Both the Single-step solution and the Numerical solution demonstrated high accuracy in estimating drug occupancy from a single PET scan [17] and showed congruence with occupancies derived from the gold standard method, the Lassen plot, applied to baseline-block scans [6, 17]. By estimating drug occupancy from a single PET scan as opposed to multiple baseline-block scans, the burden and radiation exposure on patients and staff can significantly be alleviated and arterial blood sampling reduced. Furthermore, the use of an arterial input function instead of a simplified reference tissue model facilitates the application of this displacement model in patient data. White matter abnormalities are common in brain disorders, such as epilepsy and depression [18, 19]; thus, modeling based on reference tissue, such as the centrum semiovale [7, 20, 21] might not be suitable in patients. Additionally, the displacement method could help clarify why some patients do not respond to LEV, despite relevant plasma concentrations. This could potentially refine the current clinical practices, where according to the European Medicines Agency guidelines, a typical standard dose of 500 mg LEV twice daily is gradually increased over months until the patient experiences seizure freedom or side effects, typically to a maximum dose of 1500 mg twice daily [22].

In the present study, we aimed to validate the recently published displacement model [17] utilizing the slower-acting, but widely used ASM, LEV [6], and the radioligand [11 C]UCB-J in healthy volunteers. Unlike BRIVA, LEV is by the European Medicines Agency approved for monotherapy and can also be used to treat status epilepticus [23, 24]. In the BrainDrugs Epilepsy Cohort Study [25], newly diagnosed, drug-naïve patients undergo a [11 C]UCB-J PET scan with LEV intervention and are longitudinally monitored to assess the efficacy of LEV treatment. For this reason, we here validate the displacement model in healthy individuals with LEV instead of BRIVA, and determine the plasma LEV concentration versus brain SV2A occupancy across various LEV doses. We hypothesized that (1) SV2A occupancy can be reliably estimated from a single [^11^ C]UCB-J PET displacement scan and (2) plasma LEV and brain SV2A occupancy follow a single site binding model.

## Methods

### Participants and neuroimaging

The PET scans and the T1- and T2-weighted magnetic resonance (MR) neuroimaging were acquired from 11 healthy individuals on a 3T Siemens Biograph mMR hybrid PET/MR system (Siemens Healthcare, Erlangen, Germany). All healthy individuals were scanned lying on the back with their head in a 32-channel head coil with foam supporting their head and neck.

### PET study design

Eleven healthy individuals underwent two separate 120 min. dynamic [11 C]UCB-J PET scans: In the displacement PET scan, LEV was administered approximately 60 min *after* the bolus injection of [11 C]UCB-J, whereas in the block PET scan, LEV had been administered approximately two hours *before* the bolus injection of [11 C]UCB-J. The intravenous administration (i.v.) of a LEV dose (5–30 mg/kg) was carried out over a periode of 4 min., followed by saline flush. Similarly, the i.v. administration of [11 C]UCB-J was performed as a bolus injection through a catheter placed in the elbow vein, and the PET scan was started simultaneously. Among the participants, five underwent both displacement and block PET scans on the same day, with a one-hour break in between, whereas the remaining six underwent the PET scans on separate days. Participants scanned on different days received two identical doses of LEV and approximately the same dose of [11 C]UCB-J, both administered as explain above.

The time-activity curves of the initial 60 min of the displacement PET scan, before the LEV intervention, were used to calculate the baseline SV2A binding in terms of the [^11^C]UCB-J distribution volume V_T_.

[^11^C]UCB-J was synthesized as previously described [26].

### PET processing

Dynamic PET data (256 × 256 × 207 voxels; 2.3 × 2.3 × 5 mm spatial resolution) was obtained on a 3T Siemens Biograph mMR hybrid PET/MR system (Siemens Healthcare, Erlangen, Germany). PET images were reconstructed as described previously [27]. The following frames were applied to the list-mode PET data for all displacement and block scans: 8 × 15, 8 × 30, 4 × 60, 5 × 120, 2 × 300, and 9 × 600 s for 120 min. Co-registration of PET images with structural T1 and T2 MRI scans was facilitated, and the images underwent attenuation corrected with Magnetic Resonance Attenuation Correction (MRAC) [28]. The images were motion-corrected using the AIR 5.2.5 software, which estimated a mean frame position and then resliced all frames to match it. Subsequent processing was conducted in Pvelab [29].

### Blood analyses

The radiolabeled tracer’s arterial input function, including its metabolites, was determined by manual sampling from a catheter in the radial artery during the first scan post-[^11^C]UCB-J injection at approximately 2.5, 5, 10, 25, 40, 55, 57.5, 60, 65, 70, 90, 105 and 120 min. and during the second scan at approximately 2.5, 5, 10, 25, 40, 60, 80, 100 and 120 min. An MRI-compatible autosampler (Swisstrace, Zürich, Switzerland) was used for continuous radioactivity measurements in arterial whole blood each second with a pump rate of 8 mL/min for the initial ten min. of the scans. Two technical failures with the autosampler occurred for individual 1 (second scan) and individual 8 (first scan), resulting in 8 and 10 arterial blood samples, respectively, being manually drawn during the first 2 min after [^11^C]UCB-J injection. The coincidence detector box, covered during detection, was positioned as close as possible to the individual’s arterial catheter with an approximate gap of 10 cm. Count data from the autosampler was recorded, and background radiation was subtracted. Subsequently, the data was decay corrected and calibrated using the imaging software PMOD version 3.3 (PMOD Technologies Inc.). A gamma counter (Cobra, 5003, Packard Instruments, Meriden, USA) was used to measure radioactivity from manually obtained blood samples during the scans in both whole blood and plasma. A radio-High-Performance Liquid Chromatography (HPLC) was used to determine [^11^C]UCB-J and its radioactive metabolites as previously described [30].

### Plasma levetiracetam analyses

Levetiracetam was administered through a catheter placed in an antecubital vein. The infusion of LEV was facilitated by a contrast injector with an infusion time of 4 min, followed by saline flush. Blood samples for determination of plasma LEV were collected from the arterial catheter during the first PET scan at approximately 2.5, 5, 10, 15, 30, 45, and 60 min after the LEV infusion and during the second PET scan at approximately 2.5, 5, 10, 25, 40, 60, 80, 100 and 120 min after [^11^C]UCB-J injection. For the collection of LEV samples, blood was drawn in a syringe and immediately transferred to a lithium-heparin glass without a gel tube. The sample was then gently turned approx. ten times, followed by centrifugation at 2246 G for 7 min. Plasma was transferred to cryogenic tubes and stored at -80 C until analyses.

Plasma LEV was analyzed in duplicate using the HPLC method described in the Supplementary Material and Methods section “Analysis of plasma levetiracetam concentrations”.

### Quantitative analyses

The displacement model’s theoretical framework and validation procedures and solutions have been described elsewhere [17]. Matlab code for both solutions can be accessed at https://github.com/Gjertrud/ISI. The differential equation for the 1TC displacement model was as follows:

$$\frac{d{C}_{T}\left(t\right)}{dt}={{K}_{1}C}_{p}\left(t\right)-\frac{{k}_{2}}{1+\left(1-\partial \left(t\right)\right){BP}_{ND}}{C}_{T}\left(t\right),$$

where ${C}_{T}$ is the radioligand concentration in tissue, ${C}_{p}$ is the radioligand concentration in arterial plasma, and ${BP}_{ND}=\frac{{k}_{3}}{{k}_{4}}$. $\partial \left(t\right)$ describes the time-course of SV2A occupancy by LEV.

At a given time, the delivery of the tracer into the specific compartment is defined as:

$$\left(1- \partial \left(t\right)\right)*{k}_{3}$$

For the Numerical solution,

$$\partial \left(t\right)=\left\{\begin{array}{c}0, t<{t}_{b} \\ {\partial }^{max}\left(1+2\frac{{t}_{e}-t}{{t}_{e}-{t}_{b}}\right){\left(\frac{t-{t}_{b}}{{t}_{e}-{t}_{b}}\right)}^{2}, { t}_{b}\le t<{t}_{e} \\ {\partial }^{max}, t\ge {t}_{e} \end{array}\right.$$

where ${t}_{b}$ is the time of LEV administration, and ${t}_{e}$ is the time at which the highest occupancy is reached ${(\partial }^{max})$.

${t}_{e}$ and ${\partial }^{max}$ were fitted with the constraints that ${t}_{e}>{t}_{b}$ and ${0<\partial }^{max}<1$.

For the Single-step solution (analytical solution),

$$\partial \left(t\right)=\left\{\begin{array}{c}0, t\le {t}_{s}\\ {\partial }^{max}, t>{t}_{s},\end{array}\right.$$

where ${\partial }^{max}$ is the highest occupancy and ${t}_{s}$ is the time of the step from $\partial \left(t\right)=0$ to $\partial \left(t\right)={\partial }^{max}$. Both parameters were fitted, with the constraints that ${t}_{s}>{t}_{b}$ and ${0<\partial }^{max}<1$.

Seven regional time activity curves (TACs) were generated for each individual, encompassing the following brain regions: hippocampus, frontal cortex, temporal cortex, striatum, posterior cingulate cortex, thalamus and insula. The model fits all seven TACs simultaneously, with separate values of K_1_, total distribution volume (V_T_) and fractional blood volume (v_B_) for each region, and shared non-displaceable distribution volume (V_ND_) and $\partial \left(t\right)$ across all seven regions.

The target occupancy estimates from the Single-step and the Numerical solution were compared with those obtained through the Lassen plot method [31]. For this purpose, a standard 1TCM was fitted to the first (intervention-free, approximately 60 min) part of the first scan and to the total (120 min) of the second scan. Baseline and block V_T_ values from the seven previously mentioned regions were then used to estimate the block scan occupancies with linear regression to the Lassen plot Eqs. [6, 31]:

$${V}_{T}\left(baseline\right)-{V}_{T}\left(drug\right)=occuapncy*\left({V}_{T}\left(baseline\right)-{V}_{ND}\right)$$

### Binding curves

To associate the SV2A occupancies with LEV plasma concentrations, the Emax model was used:

$$Y=\frac{Emax*X}{{IC}_{50}+X}$$

*Y* denotes the target occupancy, *Emax* denotes the maximum occupancy, *IC*_*50*_ represents the half maximal inhibitory concentration, and *X* denotes the drug concentration. *X* was computed as the area under the curve (AUC) of the plasma LEV concentration and normalized against sample duration (approximately 60 min for displacement scans and 120 min for block scans). For each participant two AUC measurements were computed. The first AUC value was computed from the start of the drug intervention to the end of the PET experiment during the first scan, and the second AUC value was computed from the entire duration of the second PET scan. The *Emax* was presented with the corresponding 95% confidence intervals (95% CI). Model estimates from the one-parameter Emax model were compared with those obtained from the two-parameter Emax model (Emax not constrained to 100%). The comparative evaluation of the two models’ fits was conducted through the extra-sum-of-squares F test.

### Statistical analyses

Data are presented as mean with a standard deviation (SD), range (minimum-maximum), 95% confidence interval (95% CI), *n* or percentage, as appropriate. The non-parametric Kruskal-Wallis test was used to test for significant differences in occupancy and V_ND_ estimates between the models. A two-sample T-test was conducted to test for significant differences in V_T_ estimates between individuals scanned twice on the same day vs. those scanned on two different days. A total sample size of 77 V_T_ estimates from 11 individuals has a statistical power of 0.8 at a statistical significance threshold of *p* ≤ 0.05 to detect group differences with a Cohen’s d effect size of 0.65. The occupancies were further compared using the Bland-Altman plot, where the x-axis represents the mean occupancy between the two methods for each individual, while the y-axis represents the absolute difference in the occupancy between the two methods for each individual. The coefficient of variation (CV) is calculated as $$\frac{SD of differences}{mean of measurements }$$* 100%. All tests were conducted with a significance level of *p* < 0.05. All statistical analyses and model fits were computed using either MATLAB version 9.13. 0 (R2022b), Natick, Massachusetts: The MathWorks Inc.; 2022 or the statistical software package R (version 386.4.1.3, R Core Team, Vienna, Austria, https://cran.r-project.org/). Model plots were generated in R or GraphPad Prism 9.0.0 (121).

## Results

### Participant demographics

The differences between baseline and postdrug V_T_ estimates were not significantly different between the five individuals scanned twice on the same day vs. the six individuals scanned on two separate days (11 ± 6.2 vs. 10 ± 3.5, respectively, *p* = 0.31, Cohen’s d = 0.17). Basic demographics and information regarding the PET experiments are presented in Table 1.

**Table 1:** Descriptive characteristics of the participants and PET experiments

	Mean ± SD or *n*	Range, min-max
**Sex (females: males)**	5:6	
**Age (years)**	37.5 ± 8.4	25–47
**Weight (kg)**	78 ± 18	50–109
**Displacement scans**		
Injected dose (MBq)	404 ± 92	195–525
Injected mass (µg)	0.35 ± 0.23	0.17–0.97
**Block scans**		
Injected dose (MBq)	337 ± 135	78–463
Injected mass (µg)	0.36 ± 0.24	0.04–0.89
**Days between scans**	29 ± 40.9	0-112
**Levetiracetam intervention in min. from PET scan start**		
**Displacement scans**	63 ± 3.7	56–67
**Block scans**	102 ± 33.1	45–136

PET: Positron emission tomography. SD: Standard deviation. *n*: Number of participants. min: Minimum. max: Maximum. Kg: Kilogram body weight on scan day. MBq: Mega becquerel. µg: Microgram

Additional information can be found in supplementary Table s1.

### SV2A occupancy

There were no significant differences in SV2A occupancies or V_ND_ estimates between the Single-step solution, the Numerical solution, or the Lassen plot (p-value = 0.55 and 0.13, respectively). All model estimates from the Numerical solution, the Single-step solution, and the Lassen plot method are presented in Table 2. A complete overview of Lassen plot parameters can be found in supplementary Table s2.

**Table 2:** SV2A occupancy estimates

		Numerical solution			Single-step solution			Lassen plot	
	LEV dose (mg/kg)	Occupancy (%)	V_ND_ (mL/cm^3^)	te (min)	Occupancy (%)	V_ND_ (mL/cm^3^)	ts (min)	Occupancy (%)	V_ND_ (mL/cm^3^)
**Mean ± SD**		74.2 **±** 14.87	4.2 **±** 1.5	19.5 **±** 9.5	69.6 **±** 14.37	4 **±** 1.7	9.3 **±** 2.5	73.3 **±** 17.93	2.9 **±** 1.2
**Individuals**									
1*	18	91.4	4.68	6	88.9	3.83	9.3	88	4.93
2*	30	87.9	4.24	12.4	82.8	3.36	7.5	87.9	2.09
**3**	27	95.8	6.62	23.7	85.5	5.95	13.2	83.4	2.28
**4**	24	81.2	2.78	15.5	75.5	1.94	9.8	92.1	0.77
**5**	30	83.5	3.55	15.1	81.6	3.21	9.7	86.7	1.78
6*	15	63	2.89	16.1	61	2.67	9.4	61.7	2.93
7*	10	57.7	2.66	14.6	57	2.62	7.4	61.2	3.84
**8**	12	76.5	6.54	21	71.7	6.02	11.7	69.6	2.43
**9**	7	55	2.77	22.5	54.9	2.94	5.5	30.5	3.18
**10**	5	56.9	5.03	43	46.4	7.43	6	69.3	4.18
11*	13	66.8	4.54	24.5	60.1	3.81	12.2	76.3	3.4

SV2A: Synaptic Vesicle Glycoprotein 2 A. LEV dose: Levetiracetam dose in milligrams per kilogram body weight. SD: Standard deviation. V_ND_: Non-displaceable distribution volume. *te*: The time where maximal occupancy is reached for the Numerical solution. *ts*: The time when the occupancy step occurs, and maximal occupancy is reached for the Single-step solution. *: Individuals scanned twice on the same day

### Estimates from the Emax model

**Fig. 1:**
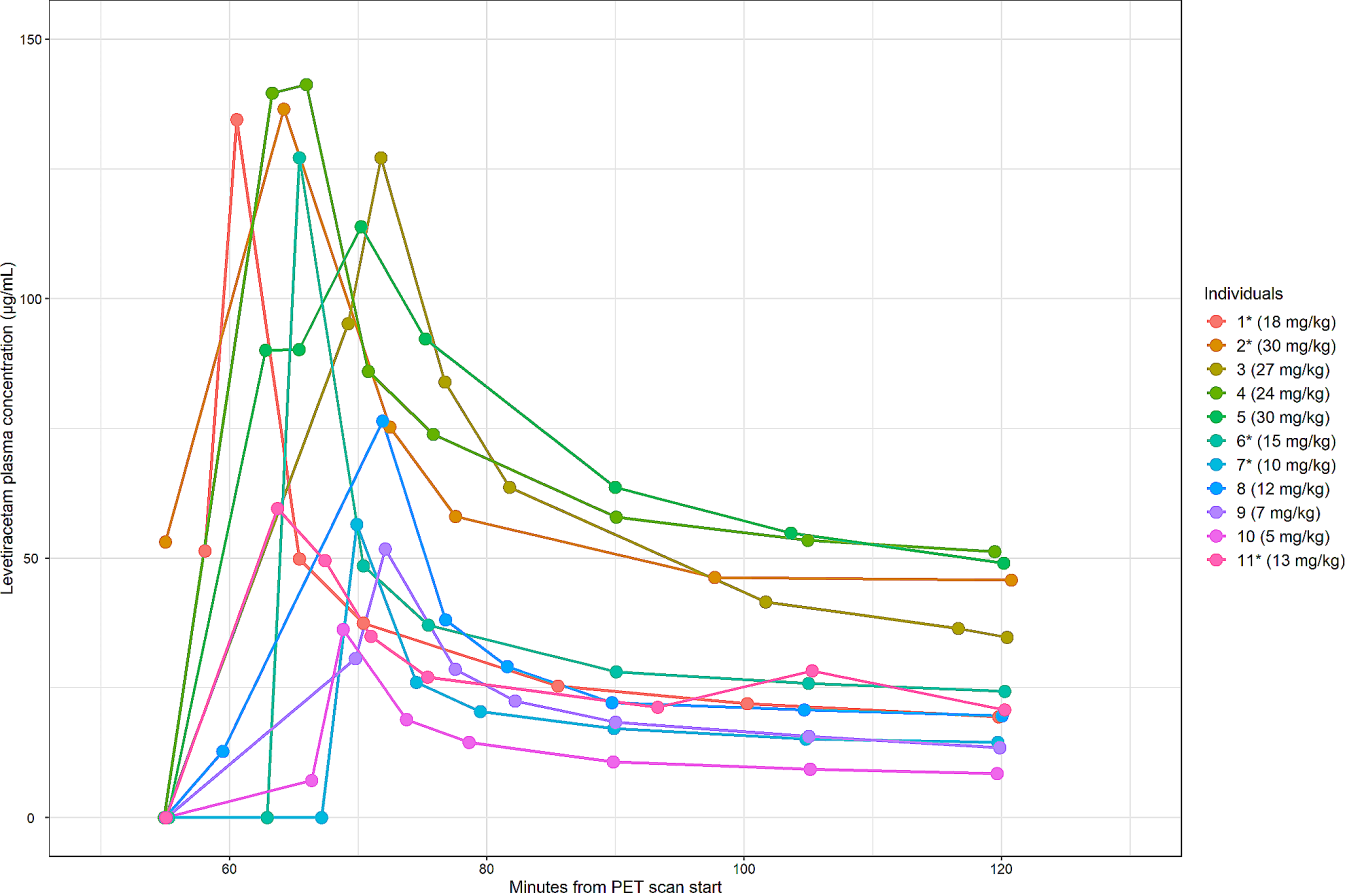
Levetiracetam plasma concentrations during displacement PET scans. * Individuals scanned twice on the same day. Mg/kg: Levetiracetam dose in milligrams per kilogram body weight. PET: Positron emission tomography

**Fig. 2:**
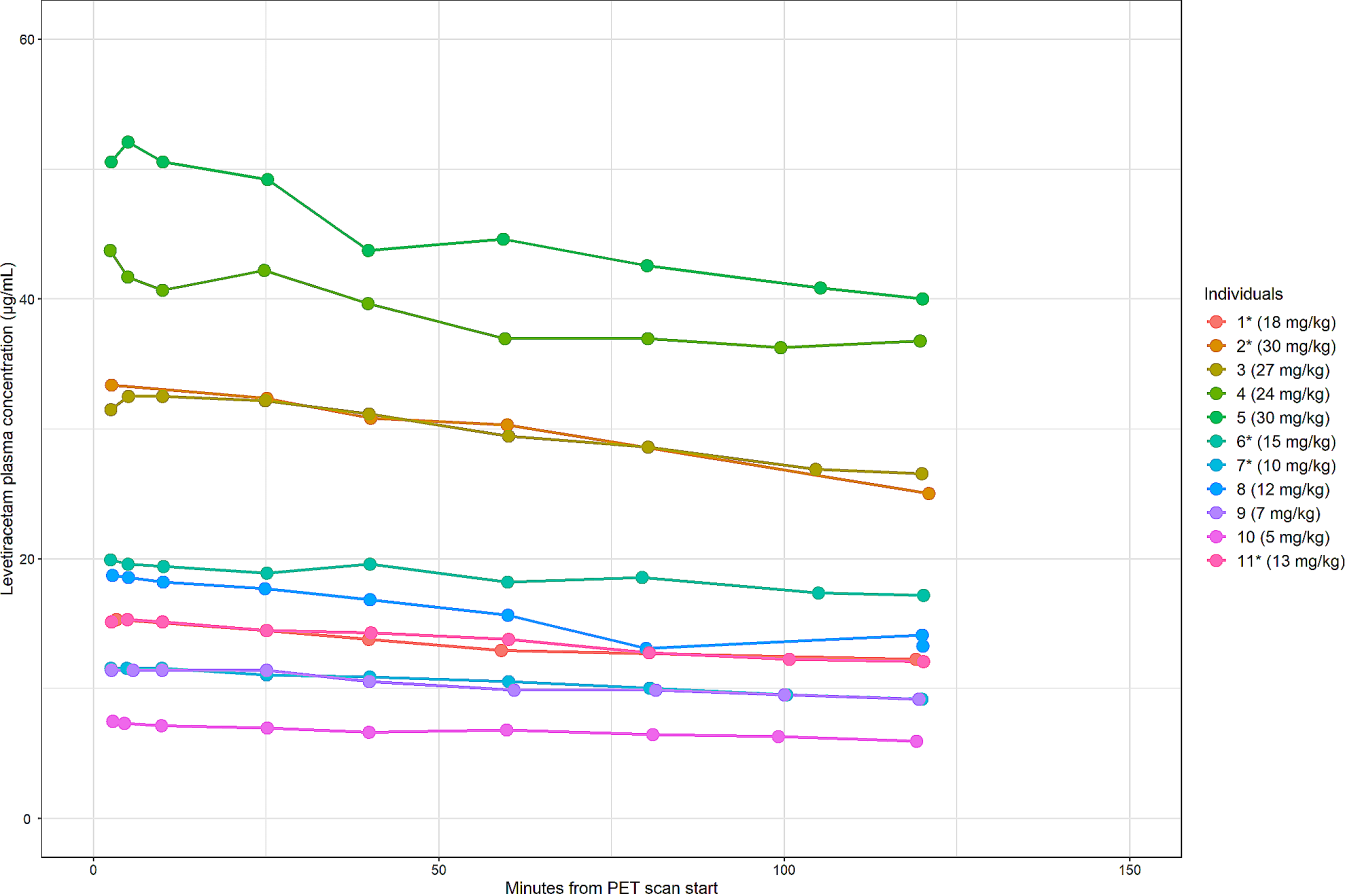
Levetiracetam plasma concentrations during block PET scans. *: Individuals scanned twice on the same day. Mg/kg: Levetiracetam dose in milligrams per kilogram body weight. PET: Positron emission tomography

The LEV doses for each participant, and their corresponding total and average AUC of the LEV plasma concentration for both the displacement and the block scans are presented in Table 3. LEV plasma concentrations during displacement and block PET scans are presented in Figs. 1 and 2, respectively.

**Table 3:** Levetiracetam dose and plasma concentration

Individuals	Levetiracetam dose (mg/kg)	Displacement scan		Block scan	
		Total AUC of LEV (µg*min/mL)	Average AUC of LEV (µg/mL)	Total AUC of LEV (µg*min/mL)	Average AUC of LEV (µg/mL)
1*	18	2123	33	1545	13
2*	30	4197	65	3504	30
**3**	27	3620	56	3479	30
**4**	24	4434	68	4504	39
**5**	30	4401	68	5230	45
6*	15	2075	32	2179	19
7*	10	1041	16	1223	11
**8**	12	1863	29	1821	16
**9**	7	1289	20	1198	10
**10**	5	735	11	773	7
11*	13	1842	28	1593	14

* Individuals scanned twice on the same day. Mg/kg: Levetiracetam dose in milligrams per kilogram body weight. AUC: Area under the plasma levetiracetam-time curve. µg: Microgram

**Fig. 3:**
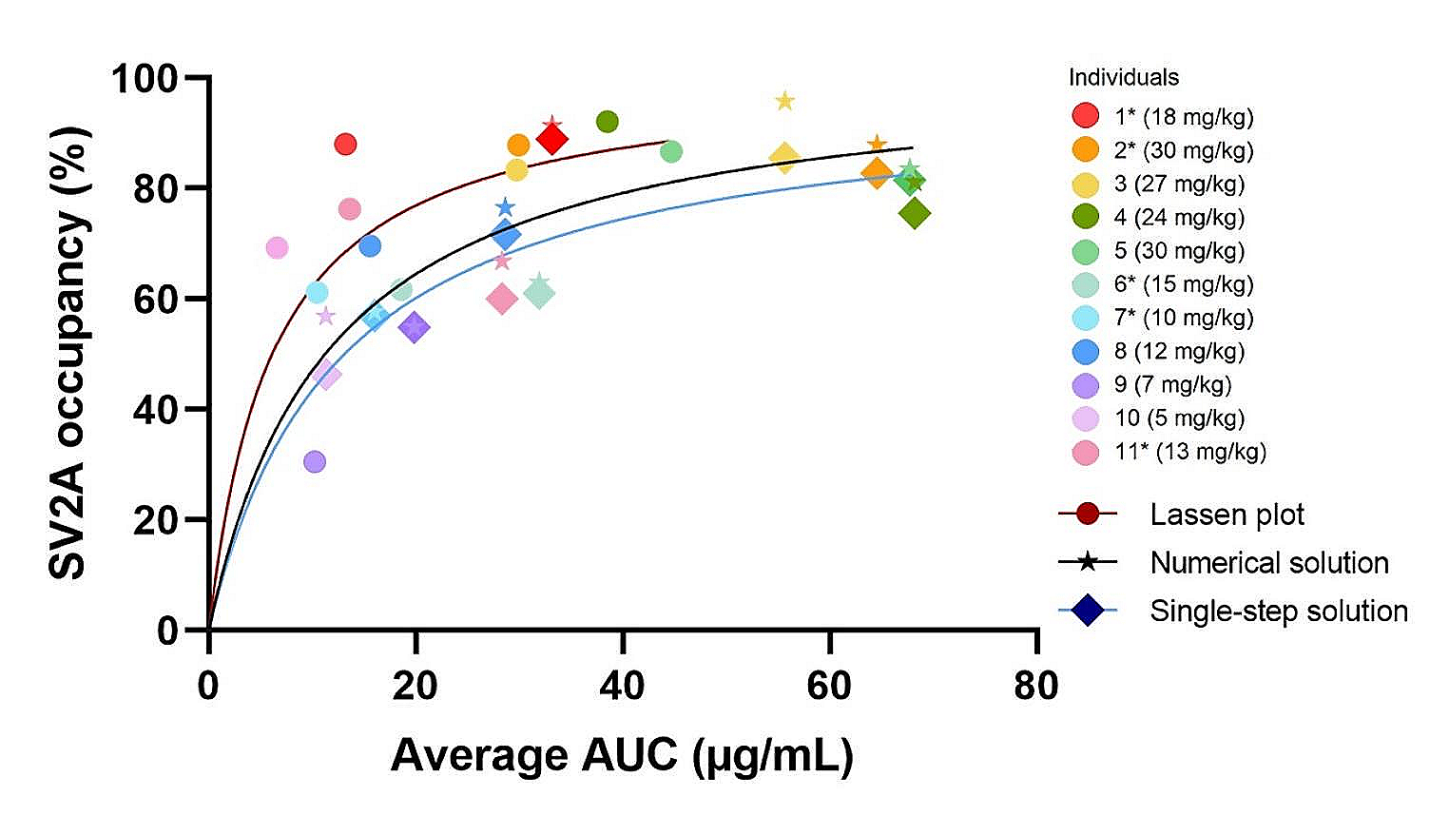
Plasma LEV concentration versus brain SV2A occupancy. Average AUC of plasma LEV concentration (µg/mL) and maximum SV2A occupancy calculated with the Lassen plot, the Numerical solution, and the Single-step solution. LEV: Levetiracetam. SV2A: Synaptic Vesicle Glycoprotein 2 A. AUC: Area under the plasma levetiracetam-time curve. *: Individuals scanned twice on the same day

Employing the two-parameter unconstrained Emax model (Eq. 1), we estimated an Emax of 97.8% (95% CI: 80–123) with a total IC_50_ of 808 µg*min/mL, average IC_50_ of 12.5 µg/mL (95% CI: 5–25) and R squared of 0.71 for the Single-step solution. The Numerical solution exhibited an Emax of 102.6% (95% CI: 84–131) with a total IC_50_ of 762 µg*min/mL, average IC_50_ of 11.8 µg/mL (95% CI: 4–25) and R squared of 0.66. The Emax for the Lassen plot method was determined to be 101% (95% CI of 71–155) with a total IC_50_ of 731 µg*min/mL, average IC_50_ of 6.3 µg/mL (95% CI: 0.08-21) and R squared of 0.4. Model plots illustrating these results are presented in Fig. 3.

Fixing Emax to 100% did not improve the model fits significantly for any of the three approaches: Single-step, Numerical, or the Lassen plot methods (p-values = 0.806, 0.81, and 0.95, respectively).

**Fig. 4:**
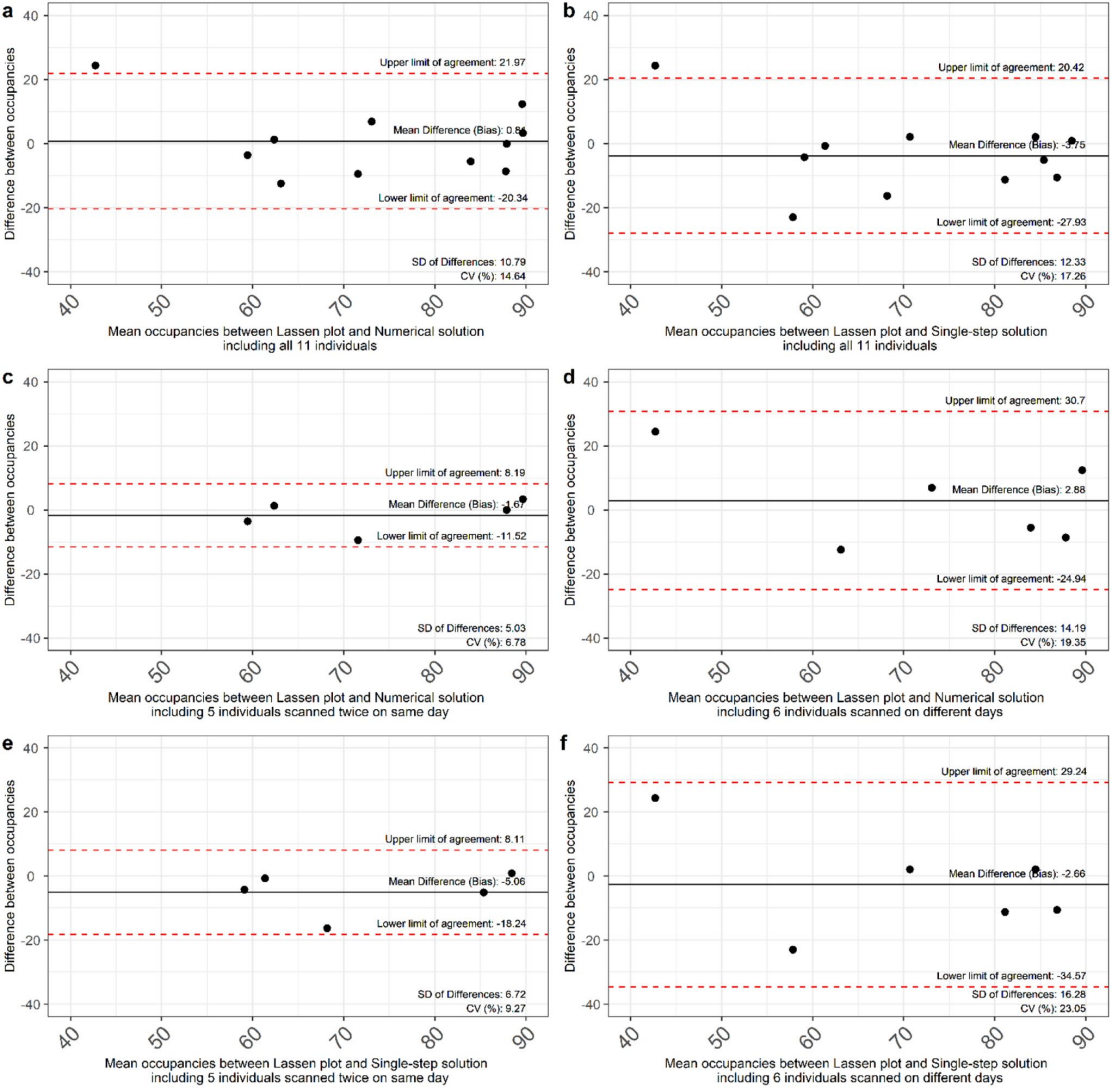
Bland-Altman comparisons between the displacement model and the Lassen plot method. Bland-Altman plots between estimated SV2A occupancies for all 11 individuals using the Numerical solution and the Lassen plot (**a**) and the Single-step solution and the Lassen plot (**b**). For individuals scanned twice on the same day (**c**) and on two different days (**d**) for the Numerical solution and the Lassen plot. For individuals scanned twice on the same day (**e**) and on two different days (**f**) for the Single-step solution and the Lassen plot. Note that the occupancy estimates from the displacement model and the Lassen plot do not necessarily correspond to the same LEV exposure. SD: Standard deviation. CV: Coefficient of variation

The three models were compared and presented in Bland-Altman plots in Fig. 4. Agreement was good, with 90.9% of data points falling within the limits of agreement for comparisons when including all eleven individuals. The CV estimates were within the same range when comparing all individuals, 14.6% vs. 17.3% for the Numerical and the Single-step solution compared with the Lassen plot (Fig. 4a and **b**). When comparing individuals scanned twice on the same day vs. individuals scanned on two different days, there was a greater variation in the CV estimates, 6.8% vs. 19.4% for the Numerical solution compared with the Lassen plot (Fig. 4c and **d**), and 9.3% vs. 23.1% for the Single-step solution compared with the Lassen plot (Fig. 4e and **f**).

## Discussion

This study presents the first human results using a newly developed displacement model for measuring drug-target engagement during a single PET scan with drug intervention [17]. Our results demonstrate the ability of this model to quantify drug occupancy from a single [^11^C]UCB-J PET scan with LEV intervention. Occupancy estimates derived from both the Single-step and the Numerical solution aligned well with those obtained through the Lassen plot method, the gold standard approach for quantifying occupancy based on V_T_ estimates from multiple PET experiments – one without drug intervention and one or more with drug intervention [7, 32]. The IC_50_ values correspond to an approximately average concentration of 12.4 µg/mL for the Single-Step solution, 11.7 µg/mL for the Numerical solution, and 6.3 µg/mL for the Lassen plot when normalized against the sample durations.

The average IC_50_ value in µg/mL, as determined using the Lassen plot method, is slightly higher compared to values presented in a previous study [6], which reported LEV IC_50_ values of 3.58, 4.02, and 4.9 µg/mL also using the Lassen plot in humans. Despite the IC_50_ value from the Lassen plot in this present study is 1.4 µg/mL higher than the highest value in the previous study [6], the values are still within the same order of magnitude. Even within the previous study [6], the IC_50_ values vary by 1.3 µg/mL. The higher IC50 value observed in the present study using the Lassen plot method could be attributed to the broader range of LEV doses administered, facilitating a 100% blockade of specific binding, in contrast to the 88% achieved in the previous study [6]. In contrast, the average IC_50_ values in µg/mL calculated from the displacement model are approximately twice as big. This might be due to LEV’s slower brain entry rate compared to BRIVA, which is approximately 30 min in humans [6]. It is possible that the steady state between LEV concentration in the brain versus plasma is not fully achieved in the latter half of the initial PET scan but is reached during the second PET scan. However, despite the difference in average IC_50_ values derived from the total AUC of plasma LEV concentrations, there was no significant difference in the estimated occupancies or V_ND_ between the displacement model and the Lassen plot. This may indicate that the displacement model handles the dynamic changes of the peak LEV plasma concentrations after the drug intervention well. The discrepancies observed in the average IC_50_ values for the displacement model vs. the Lassen plot likely stem from differences in sample durations and the timing of occupancy assessments between the displacement and the block PET scans. A way to adjust for this could be to apply a correction factor to normalize the IC_50_ to a standard duration. Regardless of this, it is noteworthy that the 95% CI for the computed average IC_50_ values overlap for the displacement model and the Lassen plot method. Additionally, even though the Lassen plot is considered the gold standard approach for calculating occupancy, new methods, such as a maximum-likelihood estimator, calculate IC_50_ better than the Lassen plot in simulated data [33]. Future PET studies could investigate the computation of occupancy with maximum-likelihood estimation vs. the displacement model potentially yielding even more comparable IC_50_ values. The well aligned IC_50_ values, expressed as total AUC and ranging from 731 to 808 µg*min/mL, for both solutions to the displacement model and the Lassen plot, indicate that the LEV concentrations are comparable between the displacement and the block scan, but the time of the drug exposure is halved for the displacement scans. Estimating IC50 values based on total AUC serves as a clinically relevant proxy for a patient’s LEV steady-state plasma concentration, as per the equation: Steady-state concentration = AUC/dosing interval. Unfortunately, to our knowledge, no other study has computed IC_50_ values for LEV using a PET displacement model, and thus the results presented in this paper cannot be compared.

Visually inspecting the three occupancy plots revealed more variability in occupancy estimates when using lower LEV doses for the Lassen plot. This trend corresponds with a previous study [17], where the displacement model performed better with higher doses of BRIVA, with the analytical Single-step solution indicating a slightly higher Emax at lower BRIVA doses.

Additionally, individual four exhibited the lowest V_ND_ of 0.77 and the highest SV2A occupancy of 92% derived from the Lassen plot method. Individual four also had relatively low V_ND_ values estimated with the Numerical and the Single-step solution, 2.78 vs. 1.94, respectively (Table 2). This individual had the longest interval between the first and the subsequent PET scan (112 days, supplementary Table s1.). Nevertheless, this participant’s body weight, injected dose of [^11^C]UCB-J, and injected mass were within the same range for both scans **(**supplementary Table s1), and the test-retest data for [^11^C]UCB-J is relatively stable over 28 days [34].

Individual nine had 40 days between the two PET scans, with comparable body weight, injected dose of [^11^C]UCB-J and injected mass **(**supplementary Table s1). An explanation for the discrepancy in the occupancy estimates between the displacement model and the Lassen plot for this individual may stem from the increased uncertainty when the models derive occupancies from lower doses of LEV. Individual nine received a LEV dose of 7 mg/kg. The same tendency is observed for individual ten, who received a LEV dose of 5 mg/kg, exhibiting a discrepancy between the occupancy estimates of 46% from the Single-step solution and 69% from the Lassen plot method. This tendency was also observed in our method paper validating the displacement model in simulated data [17]. The study presented skewed occupancies results for low doses of BRIVA for both solutions to the displacement model [17]. As previously discussed by Laurell and colleagues [17], this tendency can also be observed when applying the Lassen plot to low-dose drug administration [33]. One way to potentially improve this could involve employing a radioligand with a longer half-life, such as [^18^F]UCB-J [35]. This would extend the imaging window, ensuring sufficient radioactive tracer for drug displacement by the end of the PET scan, even with a smaller total injected dose. Future PET studies could evaluate the performance of the displacement model by comparing tracers with different half-lives.

In a previous PET study [6], four healthy individuals were i.v. administered 1500 mg LEV, resulting in occupancies ranging from 78 to 84% using the Lassen Plot method. The LEV dose, when recalculated in mg/kg from the supplementary material [6], corresponded to 18.2, 19.2, 28.6, and 25.4 mg/kg for each individual, respectively. The occupancies estimated by the displacement model in the present study, with comparable LEV doses, fell within the same range, ranging from 75 to 96%. Additionally, three healthy individuals in the same study [6] received a LEV i.v. dose of 250 and 600 mg, corresponding to a 3.6, 2.7, and 8 mg/kg dose with associated occupancies between 52 and 68%. Utilizing the displacement model, we estimated similar occupancies ranging between 46 and 57% with a LEV dose of 5 and 7 mg/kg. Naganawa and colleagues [9] estimated 76% occupancy with the Lassen plot method using 10 mg/kg i.v. LEV to block the [^11^C]UCB-J signal. In the current study, a LEV dose of 10–13 mg/kg yielded a corresponding occupancy between 57 and 77% using the displacement model. Another study [8] presented a displacement model estimating LEV occupancy from the tracer and drug rate constants as well as free fractions of [^11^C]UCB-J and the ASM and V_T_ of [^11^C]UCB-J. The displacement model initially had to fit 13 unknown parameters when computing the relationship between [^11^C]UCB-J binding and ASM uptake and clearance simultaneously [8]. Upon evaluation of the model the number of parameters was reduced to stabilize estimates including the rate constants *K*_1_ for both displacement and block PET scans, *V*_T,_ free fraction of [^11^C]UCB-J and the rate constant of the ASM ${K}_{1}^{D}$. When reanalyzing data from Finnema et al. [6], this alternative displacement model [8] estimated a mean occupancy of 84% when administering 1500 mg LEV, which is close to what we find in the present study. Thus, the displacement model presented in this paper offers a straightforward method for calculating drug occupancy, without direct estimation of drug uptake, yet giving similar occupancy results.

Finnema and colleagues have previously estimated an Emax of 88.4% for LEV, which was significantly different from 100% [6]. In the present study, multiple LEV doses were administered to explore the potential for LEV to achieve 100% blockage of specific binding. The Numerical solution yielded an estimated Emax of 102.6%, 97.8% for the Single-step solution, and 101% for the Lassen plot method. The slightly lower Emax estimated with the Single-step solution is expected, as this solution was initially developed for rapid-acting drugs where the single-step assumption is more appropriate [17]. The most prolonged *te* value for the Numerical solution was 43 min. for an individual receiving 5 mg/kg LEV, resulting in SV2A occupancies of 57%, 46%, and 69% with the Numerical solution, the Single-step solution and the Lassen plot, respectively. Unlike the pattern observed with a small dose of the fast-acting drug BRIVA [17], the Single-step solution tends to underestimate the occupancy with a low dose (5 mg/kg) of the slow-acting drug LEV when compared to the other two methods, suggesting that this model solution might be more appropriate for more rapid-acting drugsNonetheless, the application of the extra-sum-of-squares F-test revealed no significant differences when using a specific binding model with Emax constrained to 100% versus Emax unconstrained. Thus, as expected, the Single-step Emax of 97.8% was not significantly different from 100%.

Concerning SV2A-binding ASMs, LEV exhibits the slowest drug-target engagement, but whether the Numerical solution would still perform satisfactorily for even slower drugs remains to be elucidated. A potential new SV2A-binding ASM, Padsevonil, has a tenfold higher binding affinity to SV2A than BRIVA [36]. Future PET studies could investigate whether the Single-step solution performs better in estimating the drug occupancy for the rapid-acting Padsevonil relative to the Numerical solution and the Lassen plot. Notably, for the BRIVA interventions during the pig PET scans, the two distinct solutions to the displacement model yielded almost identical results [17].

A previous study in simulated and pig data [17] estimated an Emax for BRIVA to approximately 87% for both the Single-step and the Numerical solution. The study did not evaluate whether this was statistically different from 100%. In contrast, another PET study in humans [6] reported complete blocking of specific binding using BRIVA and the Lassen plot method. Based on our findings, we conclude that the displacement model shows consistent results with and performs equally well as the Lassen plot method using the more slow-acting drug LEV. Additionally, LEV can block 100% of the specific binding. In this latter study [6], LEV Emax was estimated to be 88.4% with a substantially smaller dose range. Thus, when establishing binding curves for a drug, the choice of analytical method and the dose range is important.

Previous studies have incorporated peak plasma concentrations in the Emax model due to the rapid displacement of the tracer by the drug [17]. In accordance with our expectations, we find a slower displacement of [11 C]UCB-J by LEV compared to BRIVA, and consequently, we used the AUC of the LEV plasma concentrations in the Emax model rather than the peak concentration. Notably, the AUC of the LEV plasma concentrations provided more comparable LEV concentrations between displacement and block scans, as opposed to the peak concentrations, which were much higher during the displacement scans and stable during the block scans.

Limitations of this study include considerations raised by Tuncel and colleagues, who assessed the 28 test-retest repeatability for [^11^C]UCB-J V_T_ estimates, revealing bias of -7.7 for whole brain grey matter, -1.1 for hippocampus, and − 8.2 for medial temporal lobe at retest, averaged across nine healthy individuals [34]. This potential bias could impact our results for individuals scanned on two different days, whereas the test-retest V_Ts_ for healthy individuals scanned on the same day are good, with the exception of hippocampus, which was not accounted for in the present study [37]. We did not, however, find any significant difference in V_T_ estimates from individuals scanned twice on the same day vs. individuals scanned on two different days. Additionally, both baseline and postdrug V_T_ estimates in all included regions were computed using an arterial input function and the 1TCM, which is the preferred kinetic model for [^11^C]UCB-J [7, 37]. Nevertheless, the Bland-Altman plots did reveal a greater variation in CV estimates among individuals scanned on two different days vs. individuals scanned twice on the same day. Another potential source of bias in the V_T_ estimates is that the baseline V_T_ estimates were calculated from only approximately 60 min of data, while the full 120-minute scan duration was used to calculate the postdrug V_T_ estimates for the block scan. Lastly, we would like to highlight that the occupancy estimates from the displacement model and the Lassen plot do not necessarily correspond to the same LEV exposure. As discussed above, the total AUC was computed over approx. 60 min. for the displacement scans and for the entire 120 min. of the block scans.

In conclusion, both solutions of the displacement model demonstrated comparable or improved performance compared to the Lassen plot method, with well-aligned estimations of SV2A occupancies and V_ND_ estimates. The reliable estimation of SV2A occupancy from a single [^11^C]UCB-J PET scan in the living human brain obviates the necessity for multiple scans when assessing the LEV-SV2A target engagement. The average IC_50_ values were 12.4 µg/mL for the Single-Step solution, 11.7 µg/mL for the Numerical solution, and 6.3 µg/mL for the Lassen plot. The next step is to apply this methodology in newly diagnosed patients with epilepsy from the BrainDrugs-Epilepsy cohort study [25] and investigate how SV2A-LEV target engagement is associated with LEV treatment efficacy, including seizure freedom and side effects during follow-up.

### Electronic supplementary material

Below is the link to the electronic supplementary material.

Supplementary Material 1

## Data Availability

Data is available upon reasonable request to corresponding author.
