# Supplementary material for: Assessment of cerebral drug occupancy in humans using a single PET-scan: A [11C]UCB-J PET study
Source: Eur J Nucl Med Mol Imaging. 2024 May 17;51(11):3292–304. doi: 10.1007/s00259-024-06759-x (PMC11369007; doi:10.1007/s00259-024-06759-x)
Supplement: Supplementary file 1 — Supplementary Material 1 [file 259_2024_6759_MOESM1_ESM.docx]

# **Supplementary Material and Methods**

# **Analysis of plasma levetiracetam concentrations**

## **Standards, internal standard, and quality control samples for LEV analyses**

Stock solutions (1 mg/ml) of levetiracetam (purity ≥99.9%, *Sigma-Aldrich, Denmark*) and UCB 17025 (Internal Standard, IS, kindly provided by *UCB S.A., Belgium*) were prepared in ethanol (*Region Hovedstadens Apotek, Demark*). Five calibration solutions of levetiracetam of 0, 20, 60, 100 to 200 µmol/L were prepared by diluting stock solution with distilled water (*Region Hovedstadens Apotek, Demark*) and then filling to a final volume of 100 ml with human serum free of levetiracetam. The stock and aliquots calibrator solutions were stored at −18 ◦C. A working solution of the IS (60 µg/ml) was prepared by diluting with ethanol and stored at 5◦C for maximum 6 month.

With each analytical batch 100 µl of each calibration level was prepared in the same way as a sample, analysed and used for preparation of a calibration curve. Quantification of levetiracetam in plasma or serum samples were based on the resulting five-point procedural calibration curve. Using procedural calibration will correct for overall recovery and matrix effects. In each analytical batch two spiked quality control samples were included and used for tracking the performance of the method.

## **Sample preparation**

Levetiracetam analysis was performed in double determination of centrifuged material. The procedure was in short as follows: 100 µl sample, QC sample or calibrator was diluted with 200 µl distilled water. 50 µl IS working solution, 100 µl sodium hydroxide (1 M dilution of 6,6 M sodium hydroxide, *Region Hovedstadens Apotek, Demark*) and 2,5 ml of dichloromethane (VWR, Denmark) was added. After shaking for 5 min at 600 rpm (*Ika Vibra VXR basic shaker, IKA, Germany*) the tube was centrifuged at 1467 *g* (*Hettich Rotanta 460R centrifuge, Hettich, Denmark*). The aqueous phase was removed by suction and the dichloromethane phase was frozen by submerging into dry ice in ethanol. The dichloromethane phase was transferred by pouring into a clean tube leaving behind any aqueous residue. The dichloromethane was evaporated to dryness under a stream of nitrogen at 30°C. The pellet was reconstituted in 250 µl of mobile phase, transferred to instrument vial or 96 well plates and analysed by HPLC-UV.

## **HPLC-UV method**

The analysis was performed on a Thermo Ultimate 3000 HPLC-UV system (*Thermo Scientific, Denmark*). The chromatographic separation was performed on an Acclaim PolarAdvantage II, C18, 3 µm, 120 Å, 4.6x150 mm (*Thermo Scientific, Denmark*) with a SecurityGuard Cartridge C18, 3 µm, 3x4mm (*Phenomenex, Denmark*) guard column, at 40°C. Isocratic elution was applied using a mobile phase of 85% acetonitril (HPCL-grade, *Sigma-Aldrich, Denmark*) and 15% distilled water, flowrate of 1 ml/min and 10 µl injection. Levetiracetam and IS was monitored at 220 nm. Quantification of the levetiracetam was based on bracketing procedural calibrations (linear fit, no weighting). Calibration curves were obtained by least-square regression of the peak height versus analyte concentration.

## **Method validation**

The employed method has been validated and accredited in accordance with ISO 15189 and is used for routine TDM of levetiracetam. Both plasma and serum were found to be applicable. The lower and the upper limit of quantification 10 and 700 µmol/L, respectively. the coefficient of variation for the presented method has been found to be ≤6%. The performance and precision of the method is verified by participation in the monthly external quality control program, PT-TM-AE01, provided by LGC, UK.

**Supplementary Table s1.** Detailed participant information during PET experiments.

| **Individuals** | **Displacement scan** | | | | **Block Scan** | | | | **Days between**  **scans** |
| --- | --- | --- | --- | --- | --- | --- | --- | --- | --- |
|  | **Injected dose**  **(MBq)** | **Injected dose**  **(MBq/kg)** | **Injected mass**  **(µg)** | **Body weight (kg)** | **Injected dose**  **(MBq)** | **Injected dose**  **(MBq/kg)** | **Injected mass**  **(µg)** | **Body weight (kg)** |  |
| **1*** | 195 | 2.3 | 0.4 | 84 | 338 | 4.0 | 0.5 | 84 | 0 |
| **2*** | 344 | 5.1 | 0.2 | 67 | 164 | 2.5 | 0.1 | 67 | 0 |
| **3** | 466 | 6.4 | 0.4 | 73 | 450 | 6.3 | 0.3 | 72 | 84 |
| **4** | 374 | 3.6 | 0.3 | 103 | 323 | 3.1 | 0.4 | 105 | 112 |
| **5** | 525 | 7 | 1 | 75 | 463 | 5.6 | 0.9 | 83 | 68 |
| **6*** | 365 | 7.3 | 0.3 | 50 | 381 | 7.6 | 0.5 | 50 | 0 |
| **7*** | 501 | 7.2 | 0.4 | 70 | 78 | 1.1 | 0.04 | 70 | 0 |
| **8** | 442 | 7.4 | 0.2 | 60 | 443 | 7.4 | 0.3 | 60 | 7 |
| **9** | 414 | 3.7 | 0.2 | 111 | 447 | 4.1 | 0.6 | 109 | 40 |
| **10** | 464 | 5.0 | 0.4 | 93 | 187 | 2.1 | 0.2 | 90 | 7 |
| **11*** | 353 | 5 | 0.2 | 71 | 430 | 6.0 | 0.3 | 71 | 0 |

PET: Positron emission tomography. *: Individuals scanned twice on the same day. MBq: Mega becquerel. Kg: Kilogram body weight on scan day. µg: Microgram.

**Supplementary Table s2.** Lassen plot parameters.

| **Individuals** | **LEV dose (mg/kg)** | **Slope** | **Y-intercept** | **X-intercept** | **Correlation coefficient** | **R-squared** |
| --- | --- | --- | --- | --- | --- | --- |
| **1*** | 18 | 0.88 | -4.34 | 4.93 | 0.996 | 0.99 |
| **2*** | 30 | 0.879 | -1.84 | 2.09 | 0.994 | 0.99 |
| **3** | 27 | 0.833 | -1.89 | 2.28 | 0.986 | 0.97 |
| **4** | 24 | 0.921 | -0.71 | 0.77 | 0.999 | 1 |
| **5** | 30 | 0.867 | -1.54 | 1.78 | 0.999 | 1 |
| **6*** | 15 | 0.617 | -1.81 | 2.93 | 0.985 | 0.97 |
| **7*** | 10 | 0.612 | -2.35 | 3.84 | 0.984 | 0.97 |
| **8** | 12 | 0.696 | -1.69 | 2.43 | 0.968 | 0.94 |
| **9** | 7 | 0.305 | -0.97 | 3.18 | 0.735 | 0.54 |
| **10** | 5 | 0.693 | 0.69 | 4.18 | 0.976 | 0.95 |
| **11*** | 13 | 0.763 | -2.59 | 3.39 | 0.986 | 0.97 |

*: Individuals scanned twice on the same day. LEV dose: Levetiracetam dose in milligrams per kilogram body weight.
